# The Heart of Toxicity: Details of Cardiovascular Damage Uncovered

**Published:** 2005-04

**Authors:** Bob Weinhold

In recent years, researchers have found that air pollutants can adversely affect the human cardiovascular system. But little has been uncovered about exactly how that damage occurs. German and U.S. researchers working together have now helped fill that gap, identifying for the first time several key components of heart function that are affected by air pollution **[*****EHP*** **113:440–446]**.

Cardiac arrhythmia, or a change in heart rhythm, is a major cause of death around the world. Three main factors, known as the “cardiac death triangle,” are major contributors to arrhythmias. One of these, impaired autonomic nervous system function, has been linked in numerous ways with air pollutants. This study sheds new light on links between air pollutants and the other two factors—damage to the myocardium (heart muscle) and increases in the vulnerability of the myocardium to functional alterations. The latter includes changes in the normal ebb and flow of the electrical charge around cell membranes, a phenomenon known as depolarization and repolarization.

To gather clinical data about these two factors, the team evaluated 56 East German men for the effects of selected air pollutants. The average age of the men was 66 years, and the average body mass index was 28, which is considered overweight. All of the men had stable coronary artery disease.

Using electrocardiograms (ECGs) taken every two weeks during late 2000 and early 2001, the team analyzed four key indicators of heart function and condition: QT interval duration (the total time from ventricular depolarization to complete repolarization) and T-wave amplitude, complexity, and variability of complexity (all indicators of normal ventricular repolarization). QT interval duration has been used extensively in the past to quantify repolarization, whereas T-wave data have received more acceptance only in the past few years as researchers discover their importance in elucidating nuances of heart function.

These indicators were assessed in conjunction with pollution readings taken from two outdoor monitoring stations in numerous short time intervals before the ECG readings. The pollutants studied included fine and ultrafine particulates of various compositions, nitrogen oxides, elemental and organic carbon, carbon monoxide, and sulfur dioxide. The monitoring stations were located fairly centrally to both the study center where the ECG readings were taken and the homes of the subjects, scattered around the city.

The results showed that each indicator was rapidly impacted by at least one pollutant; the significance of the impact was based on effects assessed as pollutant concentrations rose from the first to the third quartile. Particles of various sizes and makeups induced the widest-ranging effects, but nitrogen oxides, carbon monoxide, and sulfur dioxide also had an effect on certain indicators. For the three pollutants that have both German and U.S. standards—nitrogen dioxide, carbon monoxide, and sulfur dioxide—all the concentrations studied were much lower than the standard. For certain fine particulates (PM_2.5_), the third quartile was higher than the U.S. annual standard, and the maximum was higher than the U.S. annual and 24-hour standards. There are no applicable standards for the other substances evaluated.

This is the first clinical evidence of specific repolarization pathways affected by air pollutants, the authors say, and it fits observed epidemiologic patterns and speculation on biological pathways. However, the data must be viewed in light of the fact that they were derived from a high-risk group of older, overweight men with a previous history of heart disease, during a cool and humid time of year in one European setting. In addition, the pollution measurements likely didn’t fully capture the subjects’ indoor and outdoor exposures. Additional research should be able to readily address these limitations and expand on the findings.

